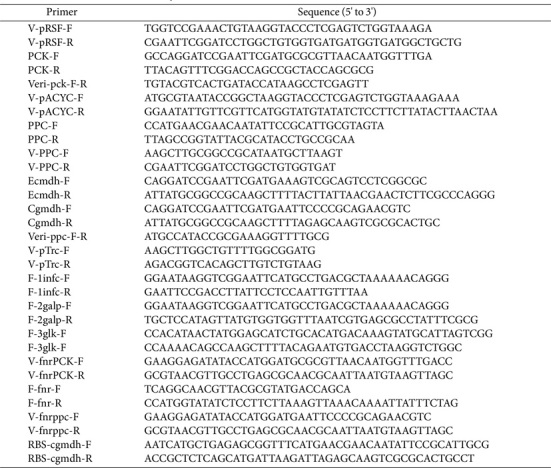# Engineering *Escherichia coli* for Anaerobic Succinate Fermentation Using Corn Stover Hydrolysate as a Substrate

**DOI:** 10.4014/jmb.2412.12041

**Published:** 2025-04-23

**Authors:** Haining Yang, Yali Dong

**Affiliations:** 1 School of Biological Engineering, Xinxiang University, Xinxiang 453003, P.R China; 2 Xinxiang University, Xinxiang 453003, P.R China; 3 National Engineering Laboratory for Cereal Fermentation Technology (NELCF), Jiangnan University, 1800 Lihu Road, Wuxi, Jiangsu 214122, P.R China

**Keywords:** Succinate, NADH, Formate dehydrogenase, isopropyl-β-d-thiogalactoside, reductive TCA pathway

## Abstract

Succinic acid is regarded as one of the most important platform chemicals used in materials science, chemistry, and food industrial applications. Currently, the main bottlenecks in the microbial succinate synthesis lie in the low titer, cofactor imbalance, and high production costs. To overcome these challenges, the reductive tricarboxylic acid cycle (TCA) and glucose uptake pathway were enhanced, increasing the titer of succinate to 4.31 g/l, 2.06-fold of the original strain. Furthermore, formate dehydrogenase from *Candida boidinii* was simultaneously overexpressed to increase the regeneration of NADH which was deficient in succinate synthesis under anaerobic condition. On this basis, the oxygen-responsive biosensor was used to replace the isopropyl-β-d-thiogalactoside (IPTG)-induction system, enabling strain to avoid the utilization of IPTG for succinate production. Using corn stover hydrolysate as the substrate, the optimum strain produced 60.74 g/l succinate in 5 L bioreactor. The engineered strain exhibited high succinate titer using biomass hydrolysate as substrate, significantly reduced the fermentation cost.

## Introduction

Succinic acid was recognized by the U.S. Department of Energy as one of 12 bio-based platform compounds for the production of high value-added products, which were widely used in the food additives, pharmaceutical and chemical industries [1, 2]. According to reports, the market for succinic acid was predicted to reach US$205.6 million by 2026, growing at a compound annual growth rate of 8.2% due to the increasing global demand for the product [1, 3]. Currently, succinate was primarily synthesized through the hydrogenation of maleic anhydride. However, this method had several disadvantages, including the use of expensive catalysts, reliance on non-renewable petroleum-based feedstocks, and cumbersome downstream processes [3]. Alternatively, microbial fermentation for producing bio-based succinic acid garnered significant attention from researchers [4].

A main greenhouse gas, CO_2_, could be fixed through reductive TCA (rTCA) pathway in anaerobic fermentation for the biosynthesis of succinic acid, which had environmental benefits [5]. It was estimated that replacing petroleum-based succinate production with the bio-based pathway could reduce greenhouse gas emissions by 5 kg CO_2_ equivalent per kg of succinic acid produced [6]. Therefore, microbial cell factories, such as *Escherichia coli* [7], *Saccharomyces cerevisiae* [8], and *Corynebacterium glutamicum* [9], and natural succinate producers, including *Anaerobiospirillum succiniciproducens* [10], *Mannheimia succiniciproducens* [11], and *Actinobacillus succinogenes* [12], were constructed, screened, and engineered be used to synthesize succinic acid. Nowadays, much effort was put into developing metabolically engineered *E. coli* strains to produce succinate due to their ease of genetic manipulation, favorable growth conditions, and good biochemical and physiological characteristics [13-15].

When using *E. coli* as host for succinate production, increasing the carbon flux to succinate production was the mainly consideration in performing metabolic engineering. To this end, lactate dehydrogenase (*ldhA*), pyruvate-formate lyase (*pflB*), and the glucose-specific PTS enzyme IICB^Glc^ of the phosphoenolpyruvate (*ptsG*) were typically knocked out to eliminate competition for reducing power and increase the metabolic flux toward succinate [15-17]. Moreover, phosphoenolpyruvate (PEP) was an indispensable precursor for succinate synthesis in the anaerobic phase [18]. PEP was converted to oxaloacetate (OAA) by either PEP carboxylase (PPC) or PEP carboxykinase (PCK). Coupled with overexpression of *ppc* on the basis of strains knocking out *ldhA*, *pflB*, and *ptsG*, Wang *et al*. developed strain SD121, which exhibited a 46.5% increase in succinate yield, reaching 1.38 mol/mol glucose, compared to the control strain SD120, which lacked *ppc* expression [19]. PCK preserved the high energy in PEP compared to PPC, thereby increasing succinate yield [20]. Tan *et al*. demonstrated that activating *ppc* expression at moderate PCK enzyme activity increased succinate titers by 66% compared to the overexpression of *ppc* alone [21]. However, the fermentation process outlined above required the addition of the inducer IPTG and used glucose as the substrate, both of which contributed to high fermentation costs. To further reduce these costs, it was necessary to modify *E. coli* to eliminate the need for IPTG and to utilize a more cost-effective carbon source for the production of succinate.

The high-levels production of succinate resulted in the rapid consumption of NADH, leading to the shortage of reducing power [22]. Therefore, the NADH regeneration pathway need to be established to supply sufficient reducing power. To this end, the formate dehydrogenase (*fdh*) was commonly used for cofactor regeneration, which can convert formate and NAD^+^ to CO_2_ and NADH, respectively [23]. For example, Yang *et al*. found that the NADH/NAD^+^ ratio of the engineered strain Mgls6-39 expressing *fdh* increased by 114.8% with the addition of 50 mM formate, leading to a 37.5% increase in succinate production [24]. Therefore, overexpression the *fdh* in succinate producing strain was critical for balancing NADH and NAD^+^.

Focusing on the challenges of unbalanced NADH and NAD^+^ and the high production cost during the anaerobic fermentation of succinate, the anaerobic succinate biosynthesis pathway was constructed and optimized coupling with the overexpression of *fdh* to increase regeneration of NADH. Furthermore, in order to reduce the fermentation cost, the oxygen-responsive biosensor was used to control the expression of succinate biosynthesis pathway, avoiding the utilization of IPTG. Finally, using corn stover hydrolysate as substrate further reduced the fermentation cost. Overall, the present study provided a feasible method for the low-cost production of succinate, contributing to the reduction of carbon emissions.

## Materials and Methods

### Strains, Medium, and Culture Conditions

*E. coli* JM109 was used as the host for plasmid cloning. *E. coli* B4 (Δ*ldhA*Δ*ptsG*Δ*poxB*, and Δ*ackA*) was used as the host strain for the constitutive expression of the target genes. The strains used in this study were listed in Table 1. Lysogeny broth (LB) was used for plasmid cloning and seed cultivation in 50 ml shake flasks. The complex medium used for succinate production in the serum bottles was supplemented with (g/l): 0.09 K_2_HPO_4_·3H_2_O, 1.14 KH_2_PO_4_, 3.0 (NH_4_)_2_SO_4_, 0.50 MgSO_4_·7H_2_O, 0.25 CaCl_2_·2H_2_O, 10 yeast extract, and 20 tryptone. In the fermentation process, rubber stoppers were added when needed to achieve anaerobic conditions. Overnight cultured seed inoculum was added to 250 ml serum bottles containing 200 ml of M9 inorganic medium (with 4 g/l glucose added). Then, the cultures were incubated at 37°C and 250 rpm until OD_600_ reached 0.6~0.8 for induction using 0.1 mM Isopropyl β-D-1-thiogalactopyranoside (IPTG). After cultivation in the aerobic phase for 24 h, the glucose was depleted. Then, the anaerobic culture was started with the supplementation of 8 g/l glucose. Kanamycin (50 μg/ml), streptomycin (50 mg/ml), and chloromycetin (25 μg/ml) were added to the media as required.

For the fed-batch fermentation, a 5 L bioreactor (Baoxing, China) was used. In the cell growth stage, fermentation was carried out at 37°C with an air-flow rate of 1 vvm and 20 g/l initial total sugar. The dissolved oxygen was kept at 30% by adjusting the agitation speed from 200 to 800 rpm. The pH was maintained at 6.80 through the automated addition of ammonia. IPTG was added into the medium once OD_600_ reached 0.8. After the initial total sugar was depleted, concentrated corn stover hydrolysate was added to the bioreactor to maintain the total sugar concentration in the fermentation broth between 5 and 20 g/l. After the cell growth reached the stationary phase, the fermentation was converted into the anaerobic condition by introducing CO_2_ into the bioreactor, allowing the engineered strain to produce succinate.

### Plasmid Construction

All plasmids and primers used in this study were listed in Tables 1 and 2, respectively. DNA polymerases and DNA seamless cloning kit were purchased from Takara (China) and Vazyme (China), respectively. The primer pair V-pCDF-F/R was used to amplify plasmid pCDF-Duet-1 to obtain V-pCDF fragment. The primer pair F-*galp*-F/R and F-*glk*-F/R were used to amplify *galp* and *glk* from *E. coli* BL21(DE3) genome. Subsequently, V-pCDF, *galp*, and *glk* were ligated and obtained the plasmid pCDF-*galp*-*glk*. The primer pair V-pacyc-F/R was used to amplify plasmid pACYC-Duet-1 to obtain V-pACYC fragment. The primer pair F-*ppc*-F/R was used to amplify *ppc* from *E. coli* BL21(DE3) genome. Subsequently, V-pACYC and *ppc* were ligated and obtained the plasmid pACYC-*ppc*. Finally, the plasmid pRSF-*pck*, pACYC-*ppc*-*mdh*, and pACYC-*ppc*-*Cgmdh* were constructed following the same procedure as described above. The *Candida boidinii* sourced *fdh* was codon optimized (The optimized sequence is shown in the Table S1), synthesized, and cloned by Azenta Life Sciences (China), obtaining plasmids of pRSF-*pck*-*fdh*. The oxygen-dependent dynamic regulation system was employed for IPTG-free succinate production. In doing this, two components of the oxygen-dependent dynamic regulatory system, the promoter P_FnrF8_ and the transcriptional regulator fnr, were amplified from the plasmid pACM4G-F8-GFP using the primer pair F-Fnr-F/R [25]. Then, the Plasmid pRSF-*pck*-*fdh* was amplified by the primer pair V-fnrPCK-F/R and ligated with the promoter P_FnrF8_, obtaining the plasmid pRSF-fnr-*pck*-*fdh*. Similarly, the plasmid pACYC-fnr-*ppc*-*Cgmdh* was constructed following the same procedure.

### Preparation Method of Corn Stover Hydrolysate

Corn stover was obtained from a village in Xinxiang (China). The preparation of corn stover hydrolysate follows the method outlined by Yang *et al*. [26]. The corn stover hydrolysate was subjected to sterilization for biosynthesis of succinate.

### Metabolite Quantification

The metabolites were analyzed by HPLC (Agilent, USA) equipped with an Aminex HPX-87H column (Bio-Rad, USA) at 50°C. The procedure was carried out as follows: the fermentation broth (1 ml) was first mixed with an equal volume of 10 mM dilute sulfuric acid. Afterward, the mixture was centrifuged at 12,000 g for 10 min, and the supernatant was subsequently filtered through a 0.22 μm filter. Metabolites in the samples such as succinic acid, lactic acid, acetic acid and formic acid were detected at 210 nm using a UV detector, while glucose and xylose were detected using a refractive index detector. Moreover, metabolites were separated using 5 mM dilute sulfuric acid at a flow rate of 0.6 ml/min with 10 μl of the detection volume.

## Results

### Enhancing the rTCA and Glucose Uptake Pathway to Boost the Succinate Production

**Fig. 1:**
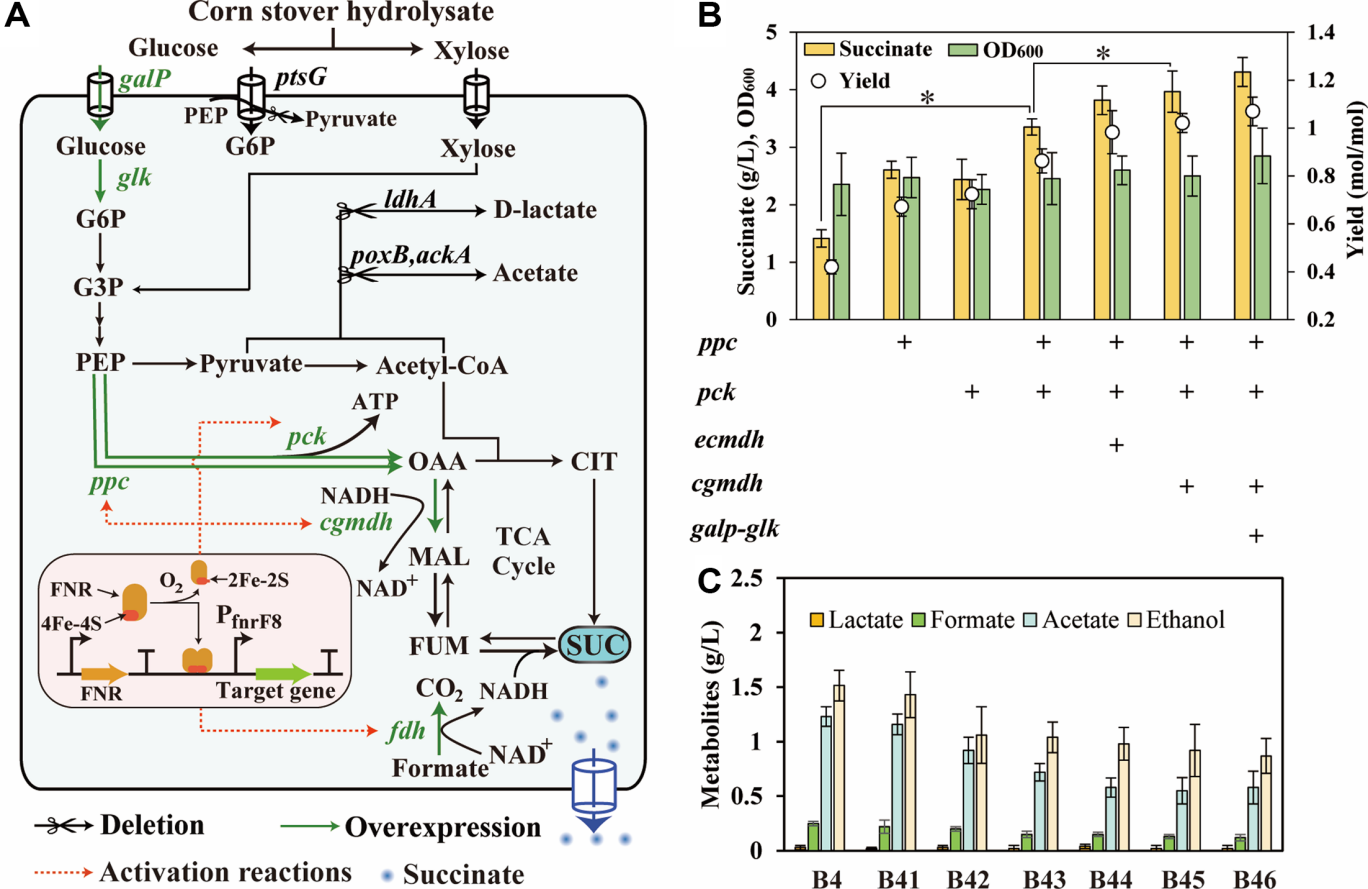
Metabolic engineering for enhancing the metabolic flux of succinate production in *E. coli* B4. (**A**) Schematic diagram of the succinate biosynthetic pathways from corn stover hydrolysate. TCA cycle, tricarboxylic acid cycle; G6P, glucose-6-phosphate; PEP: phosphoenolpyruvate; CIT, citrate; SUC, succinate; FUM, fumarate; MAL, malate; OAA, oxaloacetate; FNR, transcriptional regulator factor; The FNR dimers were activated under anaerobic conditions and subsequently activated downstream gene expression after binding upstream of the promoter P_FnrF8_. (**B**) Increase succinate titer in the anaerobic phase by overexpressing genes. +: indicates the related genes were overexpressed. (**C**) The by-products levels of succinate producing strain after combined expression of genes. All experimental data were performed in triplicate, and error bars represent the standard deviation. One-way analysis of variance (**ANOVA**) was applied to check the significance of the data (*p < 0.05).

*E. coli* B4, which deleted *ldhA*, *ptsG*, *ackA*, and *poxB*, was used as the chassis cell for succinate production. When using the rTCA pathway to produce succinate, PEP was converted to OAA via PEP carboxylase (*ppc*) or PEP carboxykinase (*pck*) (Fig. 1A). Subsequently, OAA underwent a two-step reduction reaction to produce succinate. In order to increase the titer of succinate, *ppc* and *pck* were overexpressed in *E. coli* B4 to enhance the carboxylation of PEP, resulting in *E. coli* B41 and *E. coli* B42 strains. This overexpression led to an increase in the succinate titer by 84.4% and 73.0%, respectively. The combined overexpression of *ppc* and *pck* generated *E. coli* B43 strain, producing 3.35 g/l succinate, representing a 137.6% significant increase compared to strain B4 (*p* < 0.05) (Fig. 1B).

Malate dehydrogenase (MDH) was one of the key enzymes for succinate production [4]. Therefore, *mdh* from *E. coli* or *Corynebacterium glutamicum* was overexpressed in *E. coli* B43, generating the strains *E. coli* B44 and *E. coli* B45, respectively. As a result, the succinate titers of *E. coli* B44 and *E. coli* B45 increased to 3.81 g/l and 3.98 g/l, respectively, indicating that the use of *Cgmdh* originating from *Corynebacterium glutamicum* was more efficient for converting OAA to malate (Fig. 1B). Furthermore, the absence of *ptsG* decreased the rate of glucose uptake. To accelerate glucose uptake and consumption, the galactose permease (*galp*) and glucokinase (*glk*) from *E. coli* were overexpressed in *E. coli* B45, resulting in the strain *E. coli* B46. Finally, the *E. coli* B46 strain exhibited a 4.31 g/l succinate with yield of 1.07 mol/mol glucose in shake flask, demonstrating the necessity to increase glucose uptake and consumption after the PTS was knocked out. Furthermore, with the expression of genes that enhanced the synthetic pathway of succinate, the succinate titer increased, while the titers of the by-products acetate, ethanol, and formate decreased (Fig. 1C) (The detailed by-product values were provided in Table S2).

### Enhancing the Regeneration of NADH to Improve Succinate Production

**Fig. 2:**
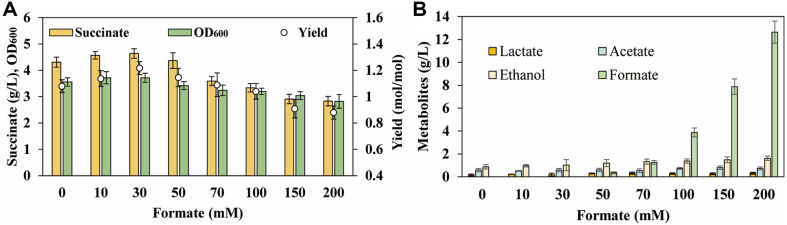
Enhancing the regeneration of NADH by overexpression of *fdh* and addition of formate. (**A**) The succinate titer and yield of *E. coli* B47 when addition of different concentrations of formate. (**B**) By-product levels of *E. coli* B47 when addition of different concentrations of formate. All experimental data were performed in triplicate, and error bars represent the standard deviation.

The shortage of NADH was the rate-limiting factor for succinate biosynthesis [27]. Therefore, in order to increase the regeneration of NADH, *fdh* was overexpressed in *E. coli* B46 to catalyze formate to CO_2_ accompanied by the conversion of NAD^+^ to NADH, generating a *E. coli* B47 strain. The results demonstrated that a continuous increase in succinate titer during the anaerobic fermentation, which correlated with a rise in the addition of formate from 0 to 30 mmol. Formate supplementation resulted in the highest succinate titer of *E. coli* B47 reaching 4.64 g/l, with a yield of 1.22 mol/mol glucose (Fig. 2A). However, subsequent elevation of formate concentrations led to heightened by-product accumulation and adversely impacted strain growth due to the presence of residual formate (Fig. 2B) (The detailed by-product values were provided in Table S3). This situation led to a decrease in succinate titer (Fig. 2A). Consequently, the addition of 30 mM formate was found to be the optimal concentration for *E. coli* B47 strain fermentation under anaerobic conditions, as it increased the availability of NADH, thereby enhancing the succinate titer.

### Reducing Fermentation Costs through the Construction of an Oxygen-Responsive Succinate Synthesis Pathway and the Utilization of Corn Stover Hydrolysate as a Substrate

**Fig. 3:**
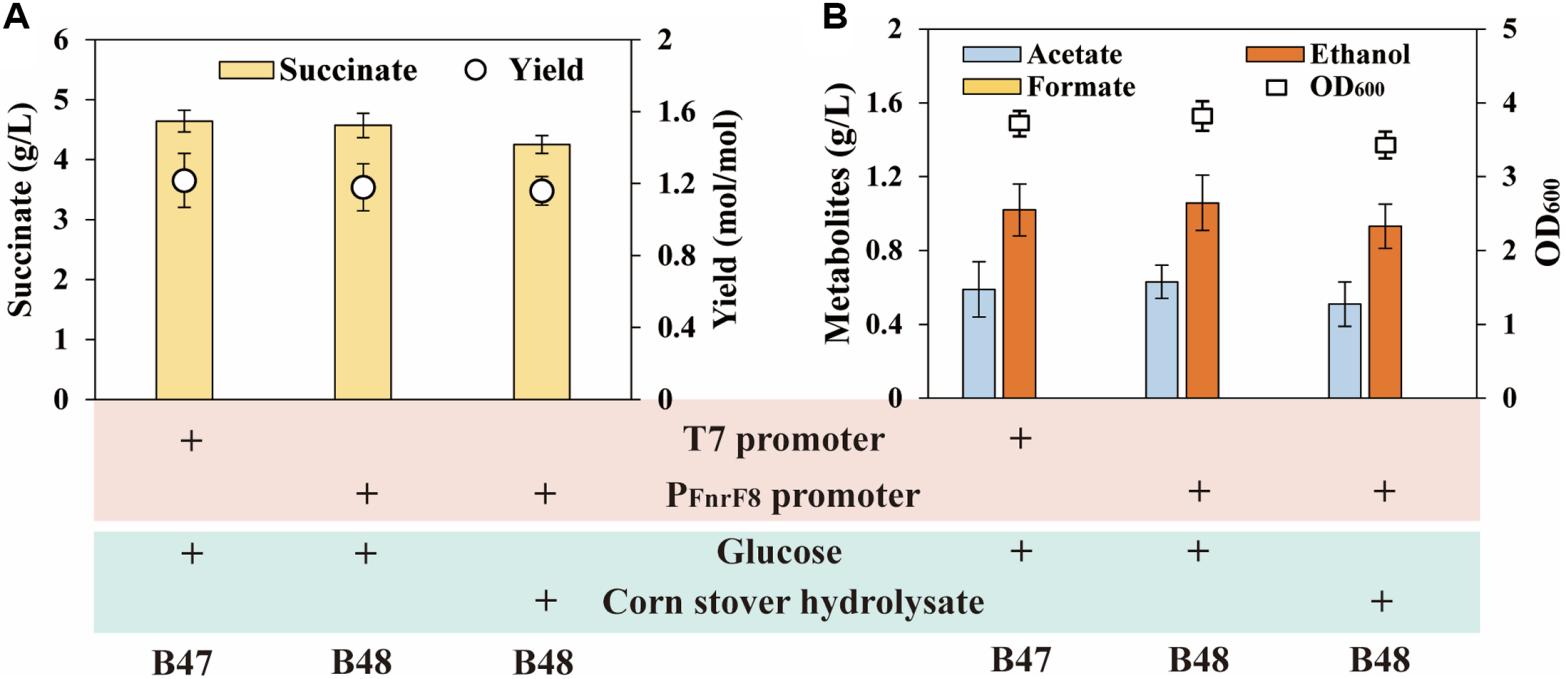
Production of succinate by *E. coli* B47 and *E. coli* B48 using glucose or corn stover hydrolysate as substrate. (**A**) The succinate titer and yield of *E. coli* B47 and *E. coli* B48 using glucose or corn stover hydrolysate. (**B**) Byproduct levels of *E. coli* B47 and *E. coli* B48 using glucose or corn stover hydrolysate. All experimental data were performed in triplicate, and error bars represent the standard deviation.

The production of succinate required the addition of the expensive inducer IPTG, thereby increasing production costs and fermentation operation complexity. To avoid the use of an inducer, an oxygen-responsive biosensor was utilized instead of the IPTG-inductive expression system to control the expression of the succinate synthetic pathway (Fig. 1A). This biosensor exhibited low transcriptional activity under aerobic conditions and increased transcriptional activity under anaerobic conditions [28] It was previously reported that the oxygen-responsive biosensor carrying FNR and promoters P_FnrF8_ generated a 6.14-fold induction change in anaerobic relative to aerobic conditions [25]. Therefore, the T7 promoters upstream of *ppc*, *Cgmdh*, *pck* and *fdh* were replaced with P_FnrF8_ to create the oxygen-inducible strain *E. coli* B48. Using the *E. coli* B48 strain for anaerobic fermentation to produce succinate resulted in 4.57 g/L of succinate without impacting the accumulation of acetate and ethanol (Fig. 3A and 3B).

To further reduce fermentation costs, corn stover hydrolysate, an inexpensive biomass feedstock, was used instead of glucose to produce succinate. Corn stover hydrolysate was added to the medium to maintain the initial total sugar concentration at 8 g/l, resulting in a titer of 4.25 g/l using the strain *E. coli* B48 as the producer (Fig. 3A). This succinate titer was slightly lower than that achieved with glucose as the substrate. Furthermore, the accumulation of by-products such as acetate and ethanol decreased compared to the utilization of glucose, likely due to the lower OD_600_. Overall, it was believed that avoiding the use of IPTG and utilizing corn stover hydrolysate as substrate for succinate production would significantly reduce the production cost of biobased succinate.

### Fed-Batch Fermentation of Succinate in 5 L Bioreactors

**Fig. 4:**
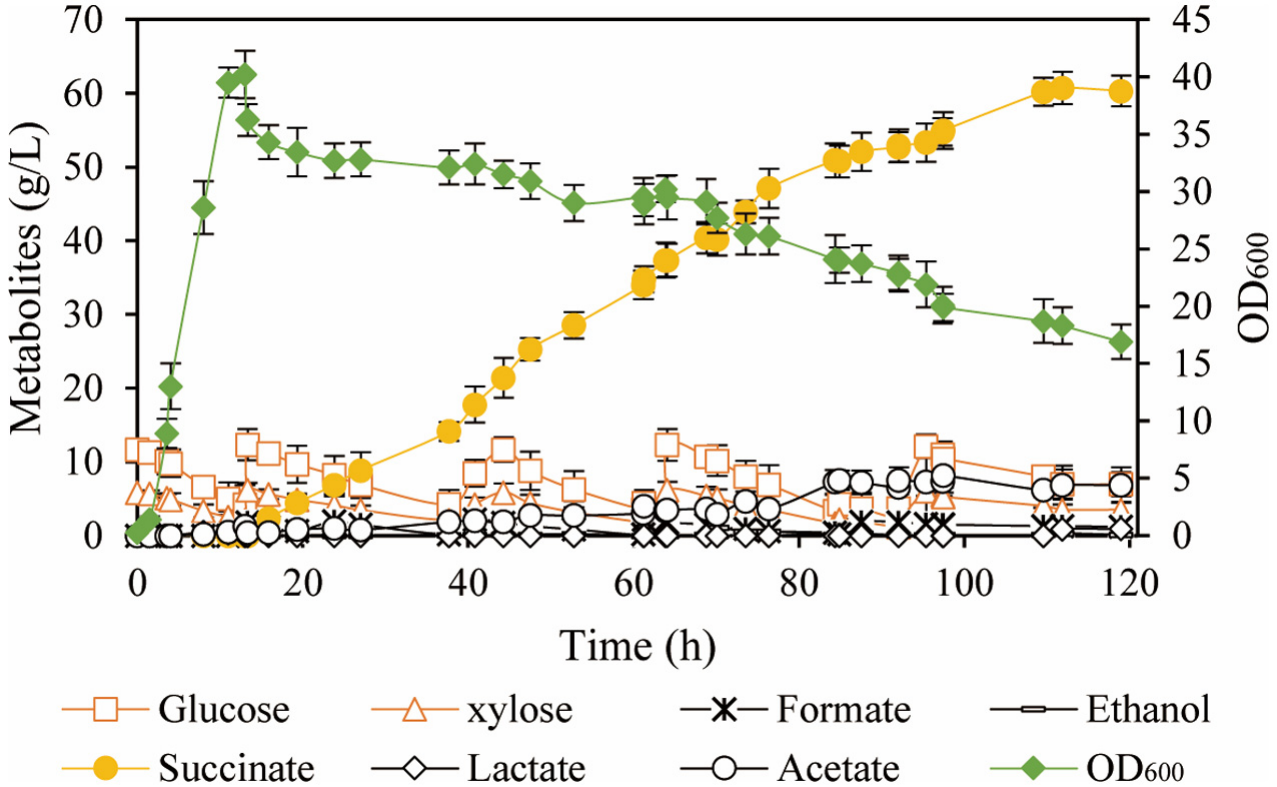
Fed-batch fermentation of *E. coli* B48 using corn stover hydrolysate as substrate.

To achieve high titers of succinate, fed-batch fermentation was employed using corn stover hydrolysate as the substrate. The initial total sugar concentration was 18.3 g/l, and the strain *E. coli* B48 reached a maximum OD_600_ of 40.2 after 13 h of aerobic fermentation. Following this, carbon dioxide was introduced into the bioreactor to initiate anaerobic fermentation for succinate production. The rapid decrease in OD_600_ observed at the beginning of the anaerobic phase likely resulted from the shift to an anaerobic environment, which adversely affected the growth of *E. coli* B48. Upon entering the anaerobic phase, the strain began producing succinate through the oxygen-responsive succinate synthesis pathway. The succinate titer reached a maximum of 60.74 g/l, with a yield of 1.42 mol/mol of total sugars after 109.6 h of fermentation (Fig. 4). However, the titer of succinate decreased during the later stages of fermentation, likely due to the declining concentration of the strain. Additionally, the by-product acetate accumulated, reaching a titer of 6.84 g/l.

## Discussion

In this study, anaerobic succinate biosynthesis pathway and glucose uptake pathway were overexpressed to enhance the metabolic flux. Subsequently, the *fdh* was overexpressed to accelerate the conversion from NAD^+^ to NADH by the addition of formate. These genetic manipulations resulted in an *E. coli* B47 that produced 4.64 g/L succinate with IPTG as inducer. To further reduce fermentation costs, an oxygen-responsive biosensor was used in place of the T7 promoter to avoid the addition of IPTG. Additionally, corn stover hydrolysate was utilized as the substrate instead of glucose. The optimal strain, *E. coli* B48, achieved a high succinate titer of 60.74 g/l in a 5 L bioreactor during fed-batch anaerobic fermentation.

During anaerobic fermentation for succinate synthesis, lactate was the predominant by-product, therefore, *ldhA* was deleted to reduce lactate production. Moreover, PEP served as an important precursor, which could be converted into OAA via the catalyzation of PPC or PCK. Comparing with PCK, PPC exhibited higher substrate affinity and catalytic velocity but consumed more energy. While, PCK can generate an ATP in the catalyzation of this reaction [29, 30]. Therefore, these enzymes exhibited complementary strengths and weaknesses in catalyzing PEP into OAA for succinate production. Our results showed that overexpression of *ppc* and *pck* increased the succinate titer by 84.4% and 73.0%, respectively, demonstrating the necessarily of enhancing this carboxylation reaction. Although the overexpression of *ppc* was more effective in improving the titer of succinate, the overexpression of *pck* also demonstrated considerable effectiveness. Tan *et al*. showed that the activity of *pck* was positively correlated with the titer of succinate [21]. Considering that ATP formation by PCK might provide more energy for cell growth, *ppc* and *pck* were co-expressed, resulting in a 137.6% increase in succinate titer. In addition, Ahn *et al*. reported that highly active *mdh* tended to increase succinate production, indicating MDH was a key enzyme in succinate production [4]. As a proof of concept, our results showing that overexpression of *Cgmdh* resulted in a 18.8% increase in succinate production (*p* < 0.05), whereas overexpression of *Ecmdh* from *E.coli* resulted in a 13.7% increase. This could be attributed to the higher activity of CgMDH under acidic or neutral conditions, which was more suitable for succinate production [4]. Overall, the overexpression and optimization of key metabolic nodes significantly improved the metabolic flux from glucose to succinate.

The deletion of *ptsG* increased the level of the succinate precursor PEP, which promoted succinate production, alleviated carbon catabolite repression (CCR), and facilitated the co-utilization of glucose and xylose [31, 32]. However, this deletion disrupted the PTS system, reducing the glucose uptake rate and consequently impairing succinate production efficiency [24]. Therefore, increasing the glucose utilization rate was essential for enhancing succinate production. Tang *et al*. demonstrated that overexpressing *galp*-*glk* in a PTS^-^ strain increased glucose utilization rate by 11.3%, which subsequently boosted succinate titer by 19.9% compared to the control [33]. This was consistent with our findings. Furthermore, the overexpression of *galp*-*glk* to enhance glucose utilization rate was also applied to the production of various other compounds, such as raspberry ketone, 2,3-butanediol, and (2S)-Naringenin [34-36]. Succinate synthesis tended to generate a significant amount of acetate as a by-product. To reduce acetate production and maximize the metabolic flux toward succinate synthesis, the *ackA* and *poxB* genes associated with acetate formation were deleted in advance. Therefore, *E. coli* B4 was employed as the chassis cell for succinate production. The experimental results showed that even after the deletion of these two genes, a small amount of acetate still accumulated in the fermentation broth (Fig. 4). This suggested that, in addition to deleting genes associated with acetate production, it was still necessary to implement a fermentation optimization strategy to further reduce acetate formation.

In the fermentation process, it was found that succinate production in shake flasks decreased after the use of oxygen-responsive biosensor to control succinate synthetic pathway (4.64 g/l *vs*. 4.57 g/l). This was possibly caused by the incomplete anaerobic conditions in the serum flask, resulting in the P_FnrF8_ promoter exhibiting only suboptimal activity, which in turn affected the succinate titer [37]. However, in bioreactor fermentation process the anaerobic condition was strictly controlled by pumping CO_2_. Therefore, this shortcoming should be avoided. Considering that the activity of P_FnrF8_ significantly influence the succinate titer, it was also possible that optimizing the activity of oxygen-responsive biosensor could further increased succinate production. Wichmann *et al*. tested a variety of oxygen-responsive biosensor and found that the promoter P_yfiD-m_ had the highest activation strength under anaerobic conditions, followed by the promoters P_FnrF8_ and P_nirB-m_ [38]. Therefore, using oxygen-responsive biosensor with varying strengths to optimize key genes in the succinate synthesis pathway was highly likely to further improved the succinate titer in the near future.

Substrate costs accounted for half of the total cost of biobased succinate production [39]. The use of inexpensive biomass hydrolysate as substrate for succinate production reduced these costs significantly [40]. Jampatesh *et al*. produced succinate using rice straw hydrolysate as a substrate, achieving a titer of 85.6 g/l with the strain *E. coli* AS1600a [41]. Similarly, Khunnonkwao *et al*. used empty oil palm fruit bunch hydrolysate as a substrate, resulting in a succinate titer of 72.45 g/l with the strain *E. coli* KJ12201-14 T [42]. Wang *et al*. employed corn stalk hydrolysate as a substrate, with the strain SD121 producing 57.81 g/l of succinate [43]. However, the yields of the aforementioned strains were relatively low, at 0.9, 0.83, and 0.87 g/g total sugar, respectively, and all required the addition of expensive inducers, increasing production costs. In contrast, the strain *E. coli* B48 achieved a yield of 0.97 g/g total sugar using corn stover hydrolysate as a substrate without the need for costly inducers. However, the succinate titer of *E. coli* strain B48 with corn stover hydrolysate as substrate was still low (60.74 g/l), and further fermentation optimization was needed to increase the succinate titer.

## Conclusion

The study improved succinate production by optimizing the reductive TCA pathway and enhancing NADH supply. Next, an oxygen-responsive biosensor was employed to enable inducer-free succinate synthesis. As a result, the final succinate titer reached 60.74 g/l in a 5 L bioreactor using corn stover hydrolysate as the substrate, without the need for IPTG induction.

## Supplemental Materials

Supplementary data for this paper are available on-line only at http://jmb.or.kr.



## Figures and Tables

**Table 1:** Strains and plasmids used in this study.

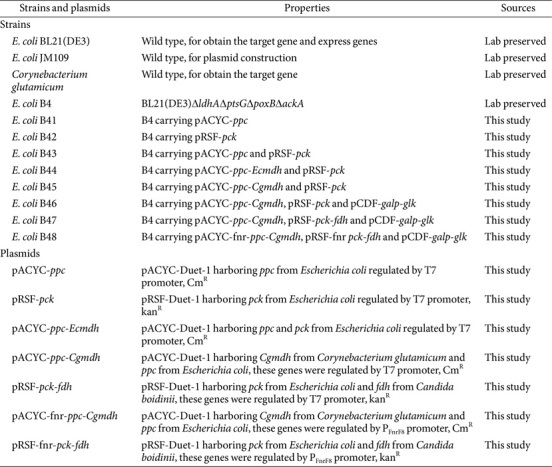

**Table 2:** Primers used in this study.