# Supplementary material for: Engineering Escherichia coli for Anaerobic Succinate Fermentation Using Corn Stover Hydrolysate as a Substrate
Source: J Microbiol Biotechnol. 2025 Apr 23;35:e2412041. doi: 10.4014/jmb.2412.12041 (PMC12089953; doi:10.4014/jmb.2412.12041)
Supplement: Supplementary file 1 [file jmb-35-e2412041-supple.pdf]

J. Microbiol. Biotechnol. 2025. 35: e2412041
https://doi.org/10.4014/jmb.2412.12041

Supplementary Tables

Engineering *Escherichia coli* for anaerobic succinate fermentation using corn stover hydrolysate as a substrate

Haining Yang[1,3,*] and Yali Dong[2]

[1]School of Biological Engineering, Xinxiang University, Xinxiang 453003, P. R. China
[2]Xinxiang University, Xinxiang 453003, P. R. China
[3]National Engineering Laboratory for Cereal Fermentation Technology (NELCF), Jiangnan University, 1800 Lihu Road, Wuxi, Jiangsu 214122, P. R. China

* Corresponding author:
Haining Yang, Email: 591096799@qq.com;

Table S1. The sequence after codon optimization.

Name	Source	The sequence after codon optimization
Fdh	*Candida boidinii*	ATGAAAATTGTGCTGGTGCTGTATGATGCGGGCAAACATGCGGCGG<br>ATGAAGAAAAACTGTATGGCTGCACCGAAAACAAACTGGGCATTG<br>CGAACTGGCTGAAAGATCAAGGCCATGAACTGATTACCACGAGCG<br>ATAAAGAAGGCGGCAACAGCGTGCTGGATCAGCATATTCCGGATGC<br>GGATATTATCATTACCACCCCGTTTCATCCGGCGTATATTACCAAAGA<br>GCGCATTGATAAAGCGAAAAAACTGAAACTGGTGGTTGTGGCGGG<br>CGTGGGCAGCGATCATATTGATCTGGATTATATTAATCAGACCGGCA<br>AAAAAATTAGCGTGCTGGAAGTGACCGGCAGCAACGTGGTGAGCG<br>TGGCGGAACATGTGCTGATGACCATGCTGGTGCTGGTGCGCAACTT<br>TGTGCCGGCGCATGAACAGATTATTAACCATGATTGGGAAGTGGCG<br>GCGATTGCGAAAGATGCGTATGATATTGAAGGCAAAACCATTGCGA<br>CCATTGGCGCGGGCCGCATTGGCTATCGCGTGCTGGAACGCCTGGT<br>GCCGTTTAACCCGAAAGAGCTGCTGTATTATGATTATCAAGCGCTGC<br>CGAAAGATGCGGAAGAAAAAGTGGGCGCGCGCCGCGTGGAAAAC<br>ATTGAAGAACTGGTGGCGCAAGCGGATATTGTGACCATTAACGCGC<br>CGCTGCATGCGGGCACCAAAGGCCTGATTAACAAAGAACTGCTGA<br>GCAAATTTAAAAAAGGCGCGTGGCTGGTGAACACCGCGCGCGGCG<br>CGATTTGCGTGGCGGAAGATGTGGCGGCCGCGCTGGAAAGCGGTC<br>AGCTGCGCGGCTATGGCGGCGATGTGTGGTTTCCGCAGCCGGCGCC<br>GAAAGATCATCCGTGGCGCGATATGCGCAACAAATATGGCGCGGGC<br>AACGCGATGACCCCGCATTATAGCGGCACCACCCTGGATGCGCAGA<br>CCCGCTATGCGGAAGGCACCAAAAACATTCTGGAAAGCTTTTTTAC<br>CGGCAAATTTGATTATCGCCCGCAAGATATTATTCTGCTGAACGGCG<br>AATATATCACGAAAGCCTATGGCAAACATGATAAAAAA

Table S2. The detailed by-product levels of the succinate-producing strain were revealed after the combined expression of genes.

	Lactate (g/L)	Formate (g/L)	Acetate (g/L)	Ethanol (g/L)
B4	0.3±0.02	0.25±0.02	1.23±0.1	1.51±0.14
B41	0.02±0.01	0.22±0.06	1.16±0.09	1.43±0.21
B42	0.03±0.02	0.2±0.02	0.92±0.12	1.06±0.26
B43	0.02±0.03	0.15±0.03	0.72±0.08	1.04±0.14
B44	0.04±0.02	0.15±0.02	0.58±0.09	0.98±0.15
B45	0.02±0.03	0.13±0.02	0.55±0.12	0.92±0.24
B46	0.02±0.03	0.12±0.03	0.58±0.15	0.87±0.16

Table S3. The detailed by-product levels of *E. coli* B47 were revealed upon the addition of different concentrations of formate.

	Lactate (g/L)	Formate (g/L)	Acetate (g/L)	Ethanol (g/L)
0	0.15±0.09	0	0.59±0.15	0.87±0.19
10	0.23±0.02	0	0.51±0.04	0.96±0.11
30	0.18±0.13	0	0.59±0.15	1.02±0.48
50	0.29±0.04	0.35±0.09	0.6±0.15	1.19±0.32
70	0.32±0.09	1.26±0.16	0.54±0.16	1.32±0.22
100	0.28±0.06	3.88±0.39	0.73±0.09	1.37±0.19
150	0.26±0.08	7.88±0.68	0.8±0.15	1.48±0.25
200	0.31±0.09	12.64±0.97	0.73±0.13	1.62±0.19
